# Supplementary figures and images for: Rab10-Positive Tubular Structures Represent a Novel Endocytic Pathway That Diverges From Canonical Macropinocytosis in RAW264 Macrophages
Source: Front Immunol. 2021 May 31;12:649600. doi: 10.3389/fimmu.2021.649600 (PMC8203412; doi:10.3389/fimmu.2021.649600)

Supplementary Figure S1

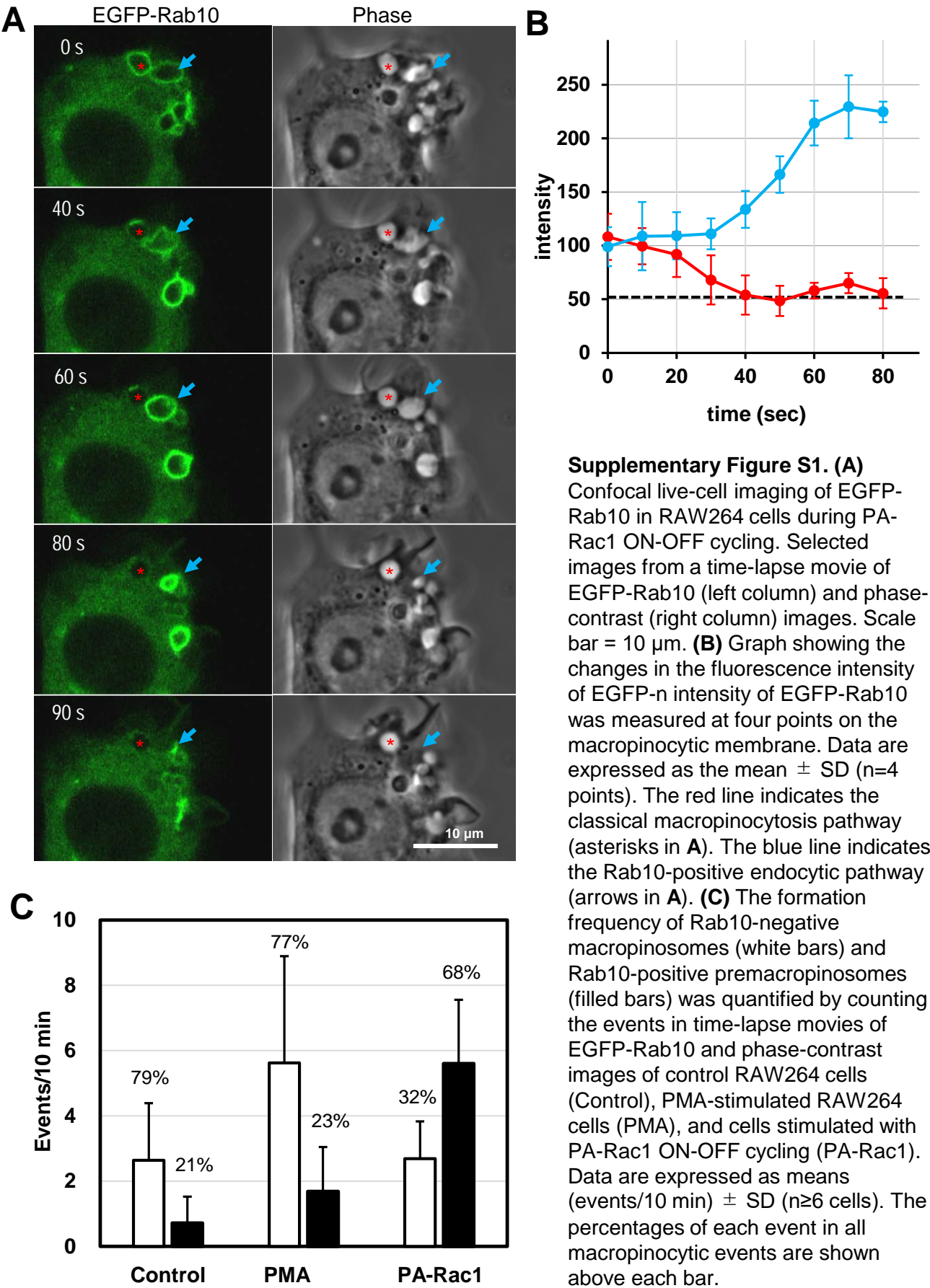

Supplement: Supplementary file 16 [file Image_1.pdf]
